# The Influence of Normative Perceptions on the Uptake of the COVID-19 TraceTogether Digital Contact Tracing System: Cross-sectional Study

**DOI:** 10.2196/30462

**Published:** 2021-11-12

**Authors:** Jeong Kyu Lee, Lavinia Lin, Hyunjin Kang

**Affiliations:** 1 Saw Swee Hock School of Public Health National University of Singapore Singapore Singapore; 2 Wee Kim Wee School of Communication and Information Nanyang Technological University Singapore Singapore

**Keywords:** COVID-19, social norms, TraceTogether, Singapore, contact tracing, mobile app, token

## Abstract

**Background:**

In 2020, the Singapore government rolled out the TraceTogether program, a digital system to facilitate contact tracing efforts in response to the COVID-19 pandemic. This system is available as a smartphone app and Bluetooth-enabled token to help identify close contacts. As of February 1, 2021, more than 80% of the population has either downloaded the mobile app or received the token in Singapore. Despite the high adoption rate of the TraceTogether mobile app and token (ie, device), it is crucial to understand the role of social and normative perceptions in uptake and usage by the public, given the collective efforts for contact tracing.

**Objective:**

This study aimed to examine normative influences (descriptive and injunctive norms) on TraceTogether device use for contact tracing purposes, informed by the theory of normative social behavior, a theoretical framework to explain how perceived social norms are related to behaviors.

**Methods:**

From January to February 2021, cross-sectional data were collected by a local research company through emailing their panel members who were (1) Singapore citizens or permanent residents aged 21 years or above; (2) able to read English; and (3) internet users with access to a personal email account. The study sample (n=1137) was restricted to those who had either downloaded the TraceTogether mobile app or received the token.

**Results:**

Multivariate (linear and ordinal logistic) regression analyses were carried out to assess the relationships of the behavioral outcome variables (TraceTogether device usage and intention of TraceTogether device usage) with potential correlates, including perceived social norms, perceived community, and interpersonal communication. Multivariate regression analyses indicated that descriptive norms (unstandardized regression coefficient β=0.31, SE=0.05; *P*<.001) and injunctive norms (unstandardized regression coefficient β=0.16, SE=0.04; *P*<.001) were significantly positively associated with the intention to use the TraceTogether device. It was also found that descriptive norms were a significant correlate of TraceTogether device use frequency (adjusted odds ratio [aOR] 2.08, 95% CI 1.66-2.61; *P*<.001). Though not significantly related to TraceTogether device use frequency, injunctive norms moderated the relationship between descriptive norms and the outcome variable (aOR 1.12, 95% CI 1.03-1.21; *P*=.005).

**Conclusions:**

This study provides useful implications for the design of effective intervention strategies to promote the uptake and usage of digital methods for contact tracing in a multiethnic Asian population. Our findings highlight that influence from social networks plays an important role in developing normative perceptions in relation to TraceTogether device use for contact tracing. To promote the uptake of the TraceTogether device and other preventive behaviors for COVID-19, it would be useful to devise norm-based interventions that address these normative perceptions by presenting high prevalence and approval of important social referents, such as family and close friends.

## Introduction

After more than a year since the World Health Organization declared COVID-19 a global pandemic, the world’s cumulative confirmed cases and deaths have reached approximately 152 million and 3.2 million, respectively [1]. In Singapore, the total number of confirmed cases has grown to 61,378, while the fatality count remains low, with 31 deaths as of May 11, 2021 [2]. Since the start of the COVID-19 outbreak, Singapore has adopted a 3-pronged approach to contain the virus by (1) reducing importation of cases through various travel restrictions and measures; (2) detecting and isolating cases early; and (3) emphasizing social responsibility and good personal hygiene practices [3].

To prevent further spread of the infectious disease, contact tracing is an evidence-based strategy for breaking the chain of transmission through the identification of close contacts who may have been exposed to the virus [4]. In Singapore, the contact tracing operation is overseen by the Ministry of Health and involves other key players including hospital staff, police, and volunteers. When a COVID-19 case is identified, the hospital staff interview the patient to map out their activity over the 14 days prior to the onset of symptoms, until diagnosis and isolation. This activity map is then submitted to the Ministry of Health, filling information gaps and identifying the patient’s close contacts. Close contacts presenting with coronavirus symptoms are hospitalized and tested for the virus, and those with no symptoms or those with low risks are placed under quarantine and phone surveillance [3]. The contact tracing operation entails extensive processes, because it requires excessive time and resources, and may involve significant errors due to recall biases, when it is done manually [5].

In an effort to augment comprehensive manual contact tracing, Singapore has launched the TraceTogether program to help identify close contacts, employing a mobile app and Bluetooth-enabled token (ie, device). Using Bluetooth technology, TraceTogether facilitates contact tracing by recording and exchanging anonymized proximity data with nearby TraceTogether devices. These Bluetooth data are encrypted, securely stored on the user’s device, and automatically deleted after 25 days. Only if the user tests positive for COVID-19 or is identified as a close contact of a positive case, the Ministry of Health asks the user to upload the data for contact tracing [6]. Although using the TraceTogether device is not compulsory, Singaporeans are encouraged to adopt the device for efficient identification and tracking of virus infection [7].

As of February 1, 2021, more than 80% of the population has either downloaded the TraceTogether app or received the token [7]. The introduction of TraceTogether, along with other digital devices, has reduced the average time required for contact tracing from 4 days to less than 1.5 days [7]. Despite the high adoption of the TraceTogether device, there is limited clarity about the uptake and usage by Singaporeans to optimize current contract tracing efforts. There is an urgent need to identify important social factors/determinants that influence TraceTogether device uptake and usage in the local context, so that health agencies and other stakeholders can develop effective campaigns and intervention strategies to increase the participation in the program.

Behavioral decisions for COVID-19 prevention, such as safe distancing and face mask wearing, are made with uncertainty. These behaviors are mainly shaped by the prominence of normative influences through direct experience or symbolically through mediated mechanisms [8]. Given that collective efforts for contact tracing are essential to contain COVID-19 transmission in the community, normative perceptions would play a significant role in the acceptance and continued use of contact tracing devices.

Since the launch of the TraceTogether program, the Singapore government has continuously emphasized to the general public the importance of contact tracing and their social responsibility to protect their community from COVID-19 [6]. For instance, the public is encouraged to check in with their TraceTogether device when visiting public venues, such as workplaces, malls, and health care facilities. There are currently several other options for checking in, which include scanning the *SafeEntry* QR code with a phone camera or a barcode on the official ID. However, these options only provide a timestamp of the date and location, without tracking the proximity with other individuals [7]. Although use of the TraceTogether device is not compulsory, the mandatory check-in at all public venues makes the public more vulnerable to normative influences [9,10]. In this regard, the theory of normative social behavior (TNSB) [11] may serve as a guiding framework to uncover mechanisms explaining the effects of perceived social norms on the uptake and usage of TraceTogether devices in Singapore.

The TNSB [10-13] is a theoretical framework to explain how and why normative influences occur. It makes a distinction between 2 closely related concepts (ie, descriptive norms and injunctive norms) and describes how these perceived norms are related to behaviors. According to the theory conceptualized by Rimal and Real, descriptive norms refer to beliefs about the prevalence of a behavior in the reference group [13]. Descriptive norms are formed largely through observations of how important referents are behaving in the group. Injunctive norms, on the other hand, refer to perceived social approval or social sanction regarding a behavior [13]. Injunctive norms help individuals to assess whether a behavior is acceptable or approved in a specific situation [12,14]. These 2 normative perceptions are considered important facilitators for the uptake of preventive measures, including the use of digital contact tracing methods.

The TNSB theorizes underlying mechanisms that explain the influence of descriptive norms on behavior by testing potential moderators, including injunctive norms, group identity, and interpersonal communication [13,15]. Individuals’ propensities to enact a behavior are significantly determined by the perception that the behavior is prevalent (eg, witnessing a group of people using the TraceTogether device for check-in at a public venue) [9,13]. The TNSB posits that perceived social pressure or approval (injunctive norms) would magnify the positive relationship between perceived prevalence and behavior [13,16]. A great deal of research has assessed the moderating role of injunctive norms in the associations between descriptive norms and various health-related behaviors, including alcohol consumption [16,17], disease screening [18], and vaccination [19,20]. Yet, the theoretical proposition has barely been tested on COVID-19–related preventive behaviors.

In addition to injunctive norms, the theory posits that the impact of descriptive norms on behavior is modified by group identity [13]. While previous studies have assessed the moderating role of group identity in the norm-behavior relationship [10,21], little is known about the role of an individual’s group identity or membership in the uptake of COVID-19–related preventive behaviors and measures. According to the theory, group identity has been conceptualized as perceived similarity with one’s reference group and aspirations to emulate members of the group [13]. Although this study focused on the psychological sense of community that is conceptually distinct from group identity, it is fruitful to examine the role of perceived community in the norm-behavior relationship for 2 reasons.

First, the TraceTogether program adopted a community-driven approach for facilitating contact tracing by exchanging anonymized proximity data with nearby TraceTogether devices [7]. Due to the importance of collective participation in the prevention of COVID-19 in the community, the TraceTogether program has been largely centered on imbuing the sense of “togetherness” or “community” among Singaporeans to promote active participation in the program. Additionally, the TraceTogether mobile app was designed to trigger feelings of closeness and connectedness to other users. For example, the app interface indicates the number of activated devices nearby and shows how many daily exchanges have been performed. As sense of community has varying definitions [22,23], in this paper, we construed it as group membership (or belonging) and emotional attachment by referencing those who are using TraceTogether devices [24]. Considering the nature of the program and the collective action for contact tracing in Singapore, strong perceptions of community would amplify the influence of normative perceptions on TraceTogether device usage for contact tracing.

Deriving from the diffusion of innovations theory [25] and social cognitive theory [26], interpersonal communication plays a crucial role in the formation of normative influences. As normative information is transmitted in a community through various forms of communication, including conversations with important social referents [15,27-29], the influence of descriptive norms on behavior would be modified by interpersonal communication. Given that the optimization of contact tracing efforts is facilitated by the collective action of the general public, normative perceptions strengthened by conversations with significant others would be a strong force motivating active participation in the TraceTogether program for contact tracing. Thus, we sought to understand the underlying process by testing the proposition that the interaction between descriptive norms and interpersonal communication is associated with TraceTogether device use. Specifically, conversing about the benefits of TraceTogether devices with important referents will magnify the norm-behavior relationship.

Studies have suggested that social norms are significant factors associated with the acceptance and intention to use contact tracing apps. However, little is known about whether and how social and normative factors influence the actual usage of a digital contact tracing device in a multiethnic population. Based on the theoretical background and abovementioned evidence, this study aimed to (1) examine whether perceived social norms (descriptive and injunctive), perceived community, and interpersonal communication influence the use of TraceTogether devices and (2) assess whether perceived injunctive norms, perceived community, and interpersonal communication moderate the relationship between perceived descriptive norms and the use of TraceTogether devices among Singapore adults who have either downloaded the TraceTogether mobile app or received the token.

## Methods

### Recruitment

From January to February 2021, cross-sectional data (N=1198) were collected by a local research company through emailing their panel members about participation in an online survey. The research company has established a panel of individuals willing to be polled on research-related topics drawn from across Singapore. The panel members are paid a sign-up fee on recruitment, and those who participate in surveys/polls are compensated with credit points by the research company in line with research industry standards. The inclusion criteria for this study were as follows: (1) Singapore citizen or permanent resident aged 21 years or above; (2) ability to read English; and (3) internet user with access to a personal email account. During the time of study recruitment, the COVID-19 situation in Singapore stabilized with only 3 clusters in the community [30,31] and a weekly moving average of less than two daily locally transmitted cases over a 2-month period [32].

Given that Singapore is a multiethnic country with 3 major Asian ethnic groups (Chinese, Malay, and Indian), the quota sampling procedure was applied to ensure that the study sample was in proportion to the demographic and ethnic structures of the national population [33]. Our study sample (n=1137) was restricted to those who had either downloaded the TraceTogether mobile app or received the token, as this study focused on the actual practice/usage of the contact tracing device (ie, use frequency or intensity) rather than the adoption of the device. All research participants were required to complete an electronic consent form prior to the online survey. Participation in this research was completely voluntary, and participants had the option to withdraw from the research at any time. To ensure the anonymity and privacy of the participants, unique study identification numbers were assigned at the beginning of the survey. Additionally, personal data (eg, identity card numbers) were not collected. The study protocol was approved by the institutional review board of Nanyang Technological University, Singapore in December 2020.

### Measures

The primary focus of the study was to examine the usage of the TraceTogether device (app/token) and its determinants. Hence, the survey questionnaire included measures for the frequency/intensity of TraceTogether device use, the intention to use the TraceTogether device, perceived social norms (descriptive and injunctive norms), perceived community, interpersonal communication, and sociodemographic characteristics. Prior to creating composite score variables for the multi-item measures derived from the TNSB (ie, perceived social norms, perceived community, and behavioral intentions), exploratory factor analyses were performed to assess the validity of the TNSB measures. Cronbach alpha was also estimated to evaluate the internal consistency (reliability) of the composite score variables.

#### Frequency/Intensity of TraceTogether Device Use

The primary outcome was assessed by the following 2 items: “In the last 7 days, how often did you use the TraceTogether app (keeping the app open in the background) when going out?” and “In the last 7 days, how often did you bring the TraceTogether token (keeping Bluetooth on) when going out?” As people tend to use either one option for the digital check-in when visiting a public venue, the intensity of TraceTogether device use was determined by the higher value of TraceTogether app use or TraceTogether token use frequency. The response options of the variable were on a 7-point scale (1, never; 2, almost never; 3, rarely; 4, sometimes; 5, quite a bit; 6, almost always; and 7, always), and they were collapsed into 3 ordinal levels (seldom [1+2+3], frequently [4+5+6], and always [7]) due to skewed distribution of the categories.

#### Intention to Use the TraceTogether Device

The secondary outcome was measured by asking how much participants agreed with 3 statements concerning their intention to use the TraceTogether device in the following week, and the responses were on a 7-point scale ranging from “strongly disagree” (1) to “strongly agree” (7). The statements were as follows: “I will use TraceTogether in the following week,” “I expect to use TraceTogether in the following week,” and “I plan to use TraceTogether in the following week.” The intention measure was adapted from the scale by Fishbein and Ajzen (Cronbach α=.96) [34].

#### Perceived Social Norms

Descriptive norms were assessed as perceived prevalence of the uptake of the TraceTogether device on a 7-point scale ranging from “not at all” (1) to “very great extent” (7). The common stem, “To what extent do you think the following groups use TraceTogether…” was completed with the following 3 items: “People like me in Singapore,” “My family,” and “My friends.” The mean of these 3 items was used as a measure of descriptive norms (Cronbach α=.93). Injunctive norms were assessed as perceived social approval on a 7-point scale ranging from “strongly disagree” (1) to “strongly agree” (7), with the statements “People like me in Singapore think that I should use TraceTogether,” “My family thinks that I should use TraceTogether,” and “My friends think that I should use TraceTogether.” The mean of the 3 items was reported (Cronbach α=.95).

#### Perceived Community

Participants’ sense of community was measured using a 5-item measure on a 7-point scale ranging from “strongly disagree” (1) to “strongly agree” (7). Adapted from the Brief Sense of Community Scale [23,35] and other community perception measures [24], the perceived community measure primarily assessed the following 2 elements of the sense of community: group membership/belonging and emotional ties with TraceTogether users. The community perception items were designed to reference TraceTogether users participating in the program. The items were “It makes me feel like I am part of a community,” “I feel a sense of attachment with other users,” “I feel emotional connection with other users,” “It reminds me of the people around me,” and “It makes me feel a sense of belonging” (Cronbach α=.95).

#### Interpersonal Communication

Interpersonal communication about the benefits of contact tracing was assessed by the average of the following 2 dichotomous items: “In the last 30 days, have you talked with your family members about the benefits of contact tracing?” and “In the last 30 days, have you talked with your friends about the benefits of contact tracing?”

In addition, data were collected on participants’ COVID-19–related worries (5 items), knowledge about contact tracing (6 items), and length of time using the TraceTogether device (1 item)*.*

Sociodemographic characteristics, including age (last birthday immediately preceding the fieldwork), gender, ethnicity, housing type, and educational attainment, were used as control variables. Data on monthly household income were excluded from the analyses due to a missing rate of 15%. Housing type and education were then used as proxies to assess the socioeconomic status. In Singapore, public housing covers over 80% of the resident population. The government has provided a public-subsidized housing scheme in which income is used to determine eligibility for subsidies for rent or purchase of an apartment unit and the size of the unit. Hence, housing type is often positively correlated with household income [36]. In addition to the demographic characteristics, participants were asked about their health status using the following item: “Would you say that in general your health is (1) poor, (2) fair, (3) good, (4) very good, or (5) excellent?” [37].

### Statistical Analysis

Numbers and percentages were used to describe participants’ characteristics that were represented by categorical variables, while means and SDs were used to describe composite score variables. Multivariate linear and ordinal logistic regression analyses were carried out to address the research aims. A multicollinearity test was performed to check if the variables were mutually correlated. Results showed that the tolerance values were greater than 0.30 and the variance inflation factors were less than 3.30, indicating that all correlates were acceptable for regression analyses. The validity of the proportional odds assumption was also assessed by the test of parallel regression assumption. The proportions of missing data were minimal, ranging from 0% to 0.01% across variables, and were handled by the listwise deletion method. All statistical analyses were performed using Stata version 17 (StataCorp).

We first performed a linear regression analysis to investigate the relationships of the intention to use the TraceTogether device with potential correlates. The analysis yielded unstandardized regression coefficients, standard errors of the unstandardized coefficients (SE), and standardized regression coefficients to indicate the strengths and directions of the associations. Next, an ordinal logistic regression analysis was carried out to identify determinants of the frequency of TraceTogether device use comprising 3 ordinal categories. The ordinal logistic regression model estimated adjusted proportional odds ratios (aORs) and 95% CIs. In these multivariate regression models, the outcome variables (ie, intention to use the TraceTogether device and use of the TraceTogether device) were regressed on the dimensions of perceived social norms (descriptive and injunctive norms), perceived community, interpersonal communication, COVID-19–related worry, and knowledge on contact tracing. Sociodemographic variables, self-assessed health status, and length of time using the TraceTogether device were modeled as control variables.

Three interaction terms were created to test the moderation effects as follows: descriptive norms × injunctive norms, descriptive norms × perceived community, and descriptive norms × interpersonal communication. The interaction terms were then added to the regression models to assess whether the relationship between the descriptive norms and outcome variables (intentions and use frequency) varied by potential moderators (ie, injunctive norms, perceived community, and interpersonal communication). This study did not adopt any procedure for model selection (eg, forward selection approach), because the study variables, including the interaction terms, were selected and entered into the regression models as guided by the theoretical framework [13].

## Results

### Sociodemographic Characteristics

Table 1 presents the sociodemographic characteristics of the study sample. Our study sample constituted 1137 participants, with a mean age of 42.9 years (SD 11.6 years). Among the participants, 583 (51.3%) were male and 584 (51.4%) had obtained a bachelor’s degree or above. The distribution of ethnicity in our study sample was as follows: 74.5% (n=847) Chinese, 15.6% (n=178) Malay, and 9.9% (n=112) Indian, which was similar to the national statistics on ethnic proportions but with a slight overrepresentation of the Indian group [33]. The majority of the participants (n=944, 83.5%) resided in public housing, while 16.5% (n=186) lived in a private property. Close to 75% (n=858) of the participants indicated their health status as “excellent,” “very good,” or “good.” Table 1 also shows the descriptive statistics of the study variables. Majority of the participants had downloaded the mobile app (n=951, 83.6%) and received the token (n=819, 72.0%). Nearly half (n=526, 46.3%) reported that they used the TraceTogether device (mobile app or token) “always” when going out in the last 7 days (ie, keeping the mobile app open in the background or keeping Bluetooth on for the token). About 22.8% (n=259) of the participants used the TraceTogether device for more than 6 months, while 16.4% (n=186) used it for less than a month.

**Table 1:** Descriptive statistics.

Variable				Value (N=1137)
**Sociodemographics**				
	**Gender, n (%)**			
		Male	583 (51.3)	
		Female	554 (48.7)	
	**Age group (years), n (%)**			
		21-34	296 (26.0)	
		35-49	517 (45.5)	
		50-64	282 (24.8)	
		65 or above	42 (3.7)	
	Mean age (years), mean (SD)		42.9 (11.6)	
	**Ethnicity, n (%)**			
		Chinese	847 (74.5)	
		Malay	178 (15.7)	
		Indian	112 (9.9)	
	**Education level, n (%)**			
		Secondary education or below	33 (2.9)	
		“A” level/diploma/polytechnic/institute of technical education/national technical certificate	513 (45.4)	
		University degree or above	584 (51.7)	
	**Housing type, n (%)**			
		1 to 3-room Housing & Development Board public housing flat	233 (20.6)	
		4-room Housing & Development Board public housing flat	440 (38.9)	
		5-room Housing & Development Board public housing flat	271 (24.0)	
		Private condo or landed house	186 (16.5)	
	**Self-assessed health status, n (%)**			
		Poor	23 (2.0)	
		Fair	256 (22.5)	
		Good	486 (42.7)	
		Very good	299 (26.3)	
		Excellent	73 (6.4)	
**TraceTogether use patterns and intentions**				
	**Adoption of the** **TraceTogether** **device, n (%)**			
		Have downloaded TraceTogether app (Yes)	951 (83.6)	
		Have received TraceTogether token (Yes)	819 (72.0)	
	**In the last 7 days, how often did you use the** **TraceTogether** **app, n (%)**			
		Never	66 (6.9)	
		Almost never	34 (3.6)	
		Rarely	56 (5.9)	
		Sometimes	136 (14.3)	
		Quite a bit	115 (12.1)	
		Almost always	159 (16.7)	
		Always	385 (40.5)	
	**In the last 7 days, how often did you bring the** **TraceTogether** **token with you, n (%)**			
		Never	89 (10.9)	
		Almost never	37 (4.5)	
		Rarely	58 (7.1)	
		Sometimes	119 (14.5)	
		Quite a bit	88 (10.7)	
		Almost always	150 (18.3)	
		Always	278 (33.9)	
	**Collapsed** **TraceTogether** **device (app or token) frequency, n (%)**			
		Never	66 (5.8)	
		Almost never	32 (2.8)	
		Rarely	49 (4.3)	
		Sometimes	136 (12.0)	
		Quite a bit	122 (10.7)	
		Almost always	206 (18.1)	
		Always	526 (46.3)	
	**How long have you used the** **TraceTogether** **app or token, n (%)**			
		Less than a month	186 (16.4)	
		1-2 months	213 (18.7)	
		2-3 months	187 (16.4)	
		3-4 months	127 (11.2)	
		4-5 months	104 (9.1)	
		5-6 months	61 (5.4)	
		More than 6 months	259 (22.8)	
	TraceTogether device use intention (mean of 3 items), mean (SD)		5.33 (1.49)	
**Correlates**				
	Descriptive norms (mean of 3 items), mean (SD)		5.17 (1.38)	
	Injunctive norms (mean of 3 items), mean (SD)		5.08 (1.48)	
	Perceived community (mean of 5 items), mean (SD)		4.65 (1.61)	
	Knowledge about contact tracing (mean of 6 items), mean (SD)		5.69 (1.16)	
	COVID-19–related worry (mean of 5 items), mean (SD)		5.23 (1.33)	
	Interpersonal communication about benefits with family members (Yes), n (%)		564 (49.6)	
	Interpersonal communication about benefits with friends (Yes), n (%)		531 (46.7)	

### Results of the Linear Regression Analysis

Table 2 displays the results of the linear regression model predicting the behavioral intention. Descriptive norms (unstandardized β=0.31, SE=0.05; *P*<.001) and injunctive norms (unstandardized β=0.16, SE=0.04; *P*<.001) were both significantly positively associated with the intention to use the TraceTogether device, such that participants who reported stronger perceptions of prevalence and social pressure were more likely to have a greater intention to use the TraceTogether device for contact tracing. As anticipated, perceived community was a significant correlate of the outcome variable, meaning that those who reported a stronger community perception had a greater intention to use the TraceTogether device (unstandardized β=0.08, SE=0.03; *P*=.001). However, the analysis did not support our anticipation of the moderating role in the relationship between descriptive norms and the intention to use the TraceTogether device. Additionally, ethnicity and length of time to adopt the TraceTogether device were significantly associated with the behavioral intention. Malay participants reported that they had a significantly lower behavioral intention than their Chinese counterparts (unstandardized β=−0.21, SE=0.09; *P*=.01). As compared with participants who used the device for less than a month, those who used the device for a longer period of time reported a higher level of intention (more than 6 months: unstandardized β=0.49, SE=0.10; *P*<.001; 3-5 months: unstandardized β=0.35, SE=0.10; *P*<.001; 1-3 months: unstandardized β=0.34, SE=0.09; *P*<.001).

**Table 2:** Linear regression analysis for correlates of TraceTogether use intention (N=1128).

Parameter			TraceTogether app/token use intention (adjusted *R*^2^=0.62)						
			Unst β^a^		St β^b^		SE^c^		*P* value
**Gender^d^ (reference: male)**									
	Female	0.07		0.02		0.06		.20	
**Age^d^ (years) (reference: 21-34)**									
	35-49	0.10		0.03		0.07		.15	
	50-64	0.12		0.03		0.08		.17	
	65 or above	0.16		0.02		0.16		.32	
**Ethnicity^d^ (reference: Chinese)**									
	Malay	−0.21		−0.05		0.09		.01	
	Indian	0.03		0.005		0.09		.79	
**Education level^d^ (reference: secondary education or below)**									
	“A” level/diploma/polytechnic/institute of technical education/national technical certificate	0.09		0.01		0.17		.60	
	University degree or above	0.08		0.03		0.06		.19	
**Housing type^d^ (reference: private condo or landed house)**									
	1 to 3-room Housing & Development Board public housing flat	0.08		0.02		0.01		.39	
	4-room Housing & Development Board public housing flat	0.05		0.02		0.08		.53	
	5-room Housing & Development Board public housing flat	0.14		0.04		0.09		.12	
Descriptive norms			0.31		0.29		0.05		<.001
Injunctive norms			0.16		0.16		0.04		<.001
Perceived community			0.08		0.09		0.03		.001
Knowledge about contact tracing			0.46		0.33		0.03		<.001
COVID-19–related worry			0.02		0.01		0.02		.48
**Interpersonal communication^d^ (reference: no)**									
	Yes	0.03		0.01		0.06		.70	
Health status			−0.05		−0.03		0.03		.11
**Length of time using the TraceTogether app token^d^ (reference: less than a month)**									
	1-3 months	0.34		0.11		0.09		<.001	
	3-6 months	0.35		0.10		0.10		<.001	
	More than 6 months	0.49		0.14		0.10		<.001	
Descriptive norms × injunctive norms			0.001		0.002		0.02		.94
Descriptive norms × perceived community			−0.03		−0.05		0.02		.08
Descriptive norms × interpersonal communication			−0.06		−0.04		0.05		.19

^a^Unst β: adjusted unstandardized regression coefficient.

^b^St β: adjusted standardized regression coefficient.

^c^SE: standard error of unstandardized regression coefficient.

^d^Gender, age, ethnicity, education, housing type, interpersonal communication, and length of time using the TraceTogether app/token were dummy-coded variables.

### Results of the Ordinal Logistic Regression Analysis

Table 3 presents the results of the ordinal logistic regression model focusing on the frequency of TraceTogether device use as an outcome. It was found that stronger perception of descriptive norms was significantly associated with elevated TraceTogether use frequency (aOR 2.08, 95% CI 1.66-2.61, *P*<.001). Contrary to our anticipation, perceived injunctive norms were not a significant correlate of the outcome variable. We also investigated the relationships of the TraceTogether device use frequency with perceived community and interpersonal communication (about benefits); however, no significant relationships were observed.

**Table 3:** Ordinal logistic regression analysis for correlates of TraceTogether use frequency (N=1128).

Parameter			TraceTogether app/token use frequency (pseudo *R*^2^=0.18)				
			Adjusted odds ratio		95% CI		*P* value
**Gender^a^ (reference: male)**							
	Female	1.05		0.82-1.35		.69	
**Age^a^ (years) (reference: 21-34)**							
	35-49	1.17		0.86-1.58		.32	
	50-64	1.08		0.74-1.58		.68	
	65 or above	1.43		0.71-2.89		.32	
**Ethnicity^a^ (reference: Chinese)**							
	Malay	0.24		0.17-0.35		<.001	
	Indian	0.66		0.43-1.02		.06	
**Education level^a^ (reference: secondary education or below)**							
	“A” level/diploma/polytechnic/institute of technical education/national technical certificate	1.48		0.70-3.18		.31	
	University degree or above	1.12		0.86-1.47		.40	
**Housing type^a^ (reference: private condo or landed house)**							
	1 to 3-room Housing & Development Board public housing flat	1.95		1.27-3.01		.002	
	4-room Housing & Development Board public housing flat	1.50		1.03-2.19		.04	
	5-room Housing & Development Board public housing flat	1.18		0.79-1.78		.42	
Descriptive norms			2.08		1.66-2.61		<.001
Injunctive norms			1.12		0.96-1.31		.15
Perceived community			1.07		0.95-1.20		.27
Knowledge about contact tracing			0.93		0.81-1.08		.34
COVID-19–related worry			1.01		0.92-1.13		.72
**Interpersonal communication^a^ (reference: no)**							
	Yes	0.82		0.61-1.09		.18	
Health status			1.02		0.88-1.19		.77
**Length of time using the TraceTogether app/token^a^ (reference: less than a month)**							
	1-3 months	3.49		2.38-5.12		<.001	
	3-6 months	4.05		2.65-6.18		<.001	
	More than 6 months	9.03		5.69-14.32		<.001	
Descriptive norms × injunctive norms			1.12		1.03-1.21		.005
Descriptive norms × perceived community			0.95		0.88-1.03		.25
Descriptive norms × interpersonal communication			0.81		0.64-1.02		.08

^a^Gender, age, ethnicity, education, housing type, interpersonal communication, and length of time using the TraceTogether app/token were dummy-coded variables.

Based on the theoretical propositions, we assessed 3 potential moderators (perceived injunctive norms, perceived community, and interpersonal communication) in the relationship between descriptive norms and TraceTogether device use frequency. The regression analysis found a significant interaction between the 2 perceived social norms (descriptive and injunctive norms) as being associated with the frequency of TraceTogether device use. This result indicated that those who reported stronger descriptive norms (ie, perception of TraceTogether use prevalence) demonstrated a significantly higher frequency of TraceTogether device use when they had stronger perception of injunctive norms (ie, social pressure to use the TraceTogether device from the members of reference groups; aOR 1.12, 95% CI 1.03-1.21; *P*=.005). However, the interactions between descriptive norms and other variables were not significant, indicating that perceived community and interpersonal communication did not moderate the influence of descriptive norms on the outcome variable.

The analysis also showed that those who adopted the TraceTogether device for a longer period of time elevated their TraceTogether device use frequency. More specifically, the odds of TraceTogether use frequency were the highest among participants who had used the device for more than 6 months (aOR 9.03, 95% CI 5.69-14.32; *P*<.001), followed by 3 to 6 months (aOR 4.05, 95% CI 2.65-6.18; *P*<.001) and 1 to 3 months (aOR 3.49, 95% CI 2.38-5.12; *P*<.001), as compared with those who had used the device for less than a month (reference category). Among sociodemographic factors, ethnicity and housing type were significantly related to the outcome variable. When comparing by ethnicity, Malay participants exhibited a 0.24 decrease in the odds of TraceTogether device use frequency (aOR 0.24, 95% CI 0.17-0.35; *P*<.001) as compared with their Chinese counterparts. In terms of housing type, those who resided in public housing, specifically in a 1 to 3-room Housing & Development Board flat (aOR 1.95, 95% CI 1.27-3.01; *P*=.002) or 4-room Housing & Development Board flat (aOR 1.50, 95% CI 1.03-2.19; *P*=.04) were more likely to use the TraceTogether device than those who lived in private properties (eg, private condo or landed house). Gender, education, age, and health status were not significantly associated with TraceTogether device use frequency.

## Discussion

### Principal Findings

Our study findings provide evidence for the normative social influence on COVID-19–related preventive behaviors in the Singapore context. Despite the high adoption rate of the TraceTogether mobile app and token in the multiethnic island state [7], actual uptake and usage are paramount to contain the spread of COVID-19. Recent studies have assessed the determinants of acceptance and behavioral intentions of mobile apps for contact tracing [38-41], and social norms have been suggested as significant factors associated with the acceptance and intention to use contact tracing apps [42]. In this study, we found direct and moderation effects of social and normative factors on the usage of a digital contact tracing device in Singapore, a multiethnic Asian city-state.

In this study, we conceptualized and assessed the following 2 distinct constructs of perceived social norms: descriptive norms (what is normal) and injunctive norms (what ought to be done) [12]. Consistent with our anticipation, use of the contact tracing device by Singaporeans was significantly influenced by descriptive norms, such that stronger perceptions of TraceTogether device use prevalence increased the intention to use the TraceTogether device and the likelihood of frequent TraceTogether device use. As discussed earlier, observable behaviors make people more vulnerable to others’ influences [43]. Use of the TraceTogether device is readily visible in Singapore as everyone is required to check-in at all public venues using the device or through other check-in methods. Our findings suggest that influence from social networks plays an important role in forming beliefs about the prevalence of TraceTogether device uptake and usage. To promote preventive behaviors, including the use of digital contact tracing methods, it would be useful to devise social norm interventions that address the target audience’s perceived descriptive norms by presenting the high prevalence of TraceTogether device use for contact tracing (or other preventive behaviors) among influential social referents, such as family and close friends [13].

To further understand the mechanisms explaining the normative influences on behavior, we assessed the influence of descriptive norms on TraceTogether device use by incorporating potential moderators, including injunctive norms, perceived community, and interpersonal communication. The study results identified that injunctive norms moderated the relationship between descriptive norms and TraceTogether device use frequency, while there were no moderation effects of perceived community and interpersonal communication. The investigation of these normative influences suggested that individual uptake of contact tracing devices is not solely governed by the perception of prevalence among referent others (eg, family and close friends). In order to boost the influence of descriptive norms, intervention messages should highlight important reference groups’ expectations and support for enacting these preventive behaviors. In this manner, the norms and expectations of the reference groups can be presented as social cues in public communications, motivating people to adopt and continuously use digital devices for contact tracing.

In this study, we also examined the role of individuals’ perceptions of community in the relationship between descriptive norms and TraceTogether device use. While the results did not support the theoretical proposition on the moderating role of perceived community, it was significantly positively associated with the behavioral intention. Although perceived community in the mobile app environment may differ from a sense of place-based community, digital contact tracing devices/apps may adopt some features to evoke feelings of belonging or emotional connection, as well as to develop affinity toward the device. The notion of community has been explored in the context of virtual communities [24,44], yet less is known about the effects and functions of perceived community in mobile and digital apps for disease prevention and health promotion. Future studies need to explore underlying mechanisms by explaining the role of perceived community in the adoption of healthy choices through the incorporation of various forms of community-induced features and functions in the apps.

### Limitations

Our study is not without limitations. First, the cross-sectional data could not be used to infer causality between perceived social norms and TraceTogether device use. Second, self-reported data used in the study may be prone to social desirability and recall biases. In addition, the data have limited generalizability to the Singapore adult population as our study sample was overrepresented with those having higher education attainment. Nevertheless, the study sample had a similar distribution of ethnicity as the national population. Despite the study limitations, this is one of the first studies to present valid evidence exploring social and normative influences on the uptake and usage of a contact tracing device for COVID-19 in a multiethnic Asian population. This study in turn contributes to the literature by serving as a baseline for future studies aimed at assessing the usage of digital devices in the COVID-19 pandemic.

### Future Research

As of June 1, 2021, the use of the TraceTogether device will become mandatory at malls, workplaces, and schools [45]. Future research may collect longitudinal data through prospective cohort studies to assess changes in social norms/social pressures and actual usage of digital contact tracing devices before and after the implementation of the new regulation. Additionally, given that Western countries have technical protocols and legal systems that differ from those in Singapore [46], population-based cross-national research may be fruitful to further understand the differences in social and environmental influences on the uptake and usage of digital contact tracing apps.

### Conclusions

To our knowledge, this is one of the first studies to explore social and normative influences on the usage of contact tracing devices for COVID-19 (TraceTogether app and token) as part of the nationwide contact tracing program in Singapore. This study provides useful information for the design of effective intervention strategies to promote the uptake of digital methods for contact tracing in the multiethnic Asian population. Our results also demonstrate that the differential functions of the 2 distinct social norm factors (descriptive and injunctive norms) are important and beneficial to curb the spread of an infectious disease in the community. The study findings suggest that influence from social networks plays an important role in forming normative perceptions (ie, perceived prevalence and social pressure) regarding TraceTogether device use for contact tracing. Therefore, it would be useful to devise norm-based interventions that harness the influence of important social referents, such as family and close friends, to promote the uptake of digital contact tracing devices and other preventive behaviors for COVID-19.

## Abbreviations

aOR: adjusted odds ratio
TNSB: theory of normative social behavior
